# TRAP1 protein signature predicts outcome in human metastatic colorectal carcinoma

**DOI:** 10.18632/oncotarget.15070

**Published:** 2017-02-03

**Authors:** Francesca Maddalena, Vittorio Simeon, Giulia Vita, Annamaria Bochicchio, Luciana Possidente, Lorenza Sisinni, Giacomo Lettini, Valentina Condelli, Danilo Swann Matassa, Valeria Li Bergolis, Alberto Fersini, Sante Romito, Michele Aieta, Antonio Ambrosi, Franca Esposito, Matteo Land riscina

**Affiliations:** ^1^ Laboratory of Preclinical and Translational Research, IRCCS, Referral Cancer Center of Basilicata, 85028 Rionero in Vulture, Italy; ^2^ Pathology, IRCCS, Referral Cancer Center of Basilicata, 85028 Rionero in Vulture, Italy; ^3^ Medical Oncology Units, IRCCS, Referral Cancer Center of Basilicata, 85028 Rionero in Vulture, Italy; ^4^ Department of Molecular Medicine and Medical Biotechnology, University of Naples Federico II, 80131 Naples, Italy; ^5^ Medical Oncology, Department of Medical and Surgical Sciences, University of Foggia, 71100 Foggia, Italy; ^6^ General Surgery Units, Department of Medical and Surgical Sciences, University of Foggia, 71100 Foggia, Italy

**Keywords:** TRAP1, client proteins, colorectal carcinoma, protein signature, overall survival

## Abstract

TRAP1 is a HSP90 molecular chaperone upregulated in colorectal carcinomas and involved in control of intracellular signaling, cell cycle, apoptosis and drug resistance, stemness and bioenergetics through co-traslational regulation of a network of client proteins. Thus, the clinical significance of TRAP1 protein network was analyzed in human colorectal cancers. TRAP1 and/or its client proteins were quantified, by immunoblot analysis, in 60 surgical specimens of colorectal carcinomas at different stages and, by immunohistochemistry, in 9 colorectal adenomatous polyps, 11 *in situ* carcinomas and 55 metastatic colorectal tumors. TRAP1 is upregulated at the transition between low- and high-grade adenomas, in *in situ* carcinomas and in about 60% of human colorectal carcinomas, being downregulated only in a small cohort of tumors. The analysis of TCGA database showed that a subgroup of colorectal tumors is characterized by gain/loss of TRAP1 copy number, this correlating with its mRNA and protein expression. Interestingly, TRAP1 is co-expressed with the majority of its client proteins and hierarchical cluster analysis showed that the upregulation of TRAP1 and associated 6-protein signature (i.e., IF2α, eF1A, TBP7, MAD2, CDK1 and βCatenin) identifies a cohort of metastatic colorectal carcinomas with a significantly shorter overall survival (HR 5.4; 95% C.I. 1.1-26.6; p=0.037). Consistently, the prognostic relevance of TRAP1 was confirmed in a cohort of 55 metastatic colorectal tumors. Finally, TRAP1 positive expression and its prognostic value are more evident in left colon cancers. These data suggest that TRAP1 protein network may provide a prognostic signature in human metastatic colorectal carcinomas.

## INTRODUCTION

Colorectal carcinoma (CRC) is the second leading cause of cancer-related death worldwide [1]. Relevant advancements in the knowledge of CRC molecular biology suggest that human CRC is a heterogeneous disease with multiple subtypes, each characterized by a specific molecular profile and a different prognosis [2, 3]. However, besides these achievements, several attempts are still ongoing to validate novel biomarkers and gene signatures to select CRC patients with different clinical behavior, in order to tailor therapies on the molecular profile of each tumor and design personalized treatments [4].

TRAP1 is a HSP90 molecular chaperone upregulated in several human malignancies [5, 6] and, among others, human CRC [7]. High TRAP1 levels have been proposed as prognostic biomarker in this malignancy, being associated with extensive lymph node dissemination [8] and, together with high ERCC1, with poor overall survival (OS) in metastatic disease [9]. TRAP1 is responsible for several functions of cancer cells, i.e. protection from stress and apoptosis, drug resistance, protein homeostasis, maintenance of stemness, intracellular signaling, cell migration, cell cycle regulation, and bioenergetics [5, 7, 10–19]. TRAP1 regulation of these cell functions depends on the co-translational quality control of specific client proteins and, thus, the modulation of the expression/ubiquitination of a network of proteins [11, 12]. In such a context, we previously suggested that human CRCs with increased TRAP1 expression are characterized by selective upregulation of specific TRAP1 network proteins [6, 13, 20, 21], even though the clinical significance of this regulation is still largely unexplored [6]. Thus, this study was designed to obtain a comprehensive evaluation of the biological and clinical relevance of TRAP1 protein network in human CRC and to address the hypothesis that the coordinated upregulation of TRAP1 and its client proteins may serve as a prognostic signature in metastatic disease.

## RESULTS

### TRAP1 upregulation is an early event in human colorectal carcinogenesis and is partially dependent on transcriptional mechanisms

**Figure 1:**
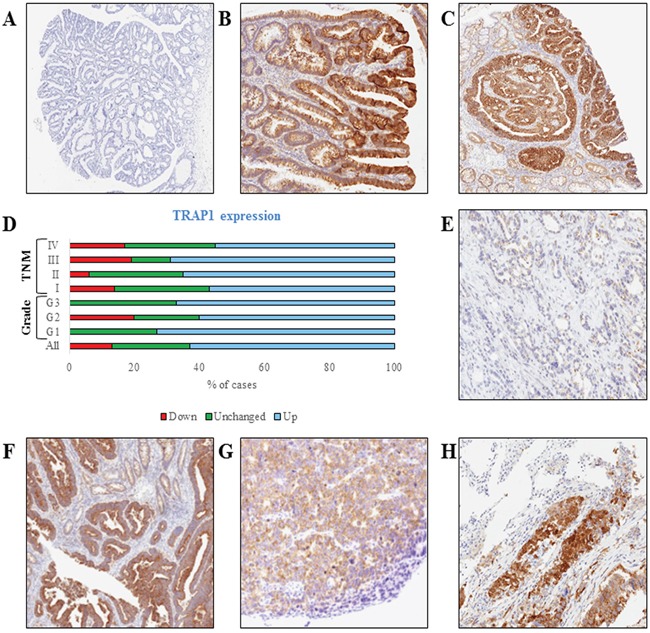
TRAP1 is upregulated in high-grade colon adenomas and in the majority of human colorectal carcinomas **A-C**. TRAP1 IHC staining in representative cases of low-grade (A, Magnification 2x) and high-grade (B, 4x) adenomatous polyps and *in situ* carcinoma (C, 4x). **D**. Bar graph reporting percentages of TRAP1 protein expression in 60 cases of human CRCs and its distribution according to tumor grade and TNM stage. **E-H**. TRAP1 IHC staining in representative cases of TRAP1-negative (E, 10x) and TRAP1-positive (F, 5x) primary colorectal carcinomas and in lymph node (G, 10x) and lung (H, 10x) metastases from a TRAP1-positive colorectal carcinoma.

To address the relevance of TRAP1 in colorectal carcinogenesis, its expression was analyzed, by immunohistochemistry, in 9 colon adenomatous polyps with different grading and 11 *in situ* carcinomas. Interestingly, TRAP1 is upregulated at the transition between low- and high-grade adenomas, being the latter characterized by increased levels of TRAP1 in 4/6 cases (Figure 1A–1B) and is upregulated in 7/11 *in situ* carcinomas (Figure 1C). TRAP1 expression was further evaluated, by immunoblot analysis, in 60 human CRCs at different stages and in the respective non-infiltrated normal colon mucosa (study cohort – Table 1). Indeed, TRAP1 upregulation was observed in 63% of cases, being its expression unchanged or downregulated in, respectively, 24% and 13% of tumors (Figure 1D–1F and Supplementary Figure 1). No correlation was observed between TRAP1 expression and TNM stage, tumor grade and lymph node dissemination (Figure 1D and Supplementary Table 1). In addition, TRAP1 protein expression was analyzed, by immunohistochemistry, in selective TRAP1-positive tumors in comparison with respective lymph node and distant metastases and no major differences were observed (Figure 1G–1H).

**Table 1:** Demographic and clinicopathological characteristics of patients

**Characteristics**	**No of patients (%)**	
**Adenomatous polyps**	9	
***In situ*** **carcinomas**	11	
**Colorectal carcinomas - Study cohort**	60	
**Colorectal carcinomas - Validation cohort**	55	
	**Study Cohort**	**Validation Cohort**
**Sex, n (%)**		
- Male	38 (63)	39 (71)
- Female	22 (37)	16 (29)
**Age (years)**		
- Mean ± SD	72.7±9.2	68.8 ± 7.9
- Median (range)	72.5 (54-89)	70 (39-80)
**TNM Stage, n (%)**		
- I	8 (13.3)	-
- II	20 (33.4)	-
- III	14 (23,3)	-
- IV	18 (30.0)	55 (100)
**Grading, n (%)**		
- G1	11 (18.3)	8 (15)
- G2	40 (66.7)	37 (67)
- G3	9 (15.0)	10 (18)
**Tumor Site**		
- Right/transverse colon	22 (37)	10 (18)
- Left colon	35 (58)	40 (73)
- Not reported	3 (5)	5 (9)
**Metastatic pattern, n (%)**		
- Liver	17 (28)	29 (53)
- Lung	8 (13)	23 (42)
- Peritoneum	6 (10)	6 (11)
- Other sites	3 (5)	3 (6)
**Adjuvant chemotherapy (TNM stage II/III, %)**		
- Yes	15 (42)	
- No	21 (58)	
**First line chemotherapy (TNM stage IV, %)**		
- Yes	15 (83)	
- No	3 (17)	55 (100)

**Figure 2:**
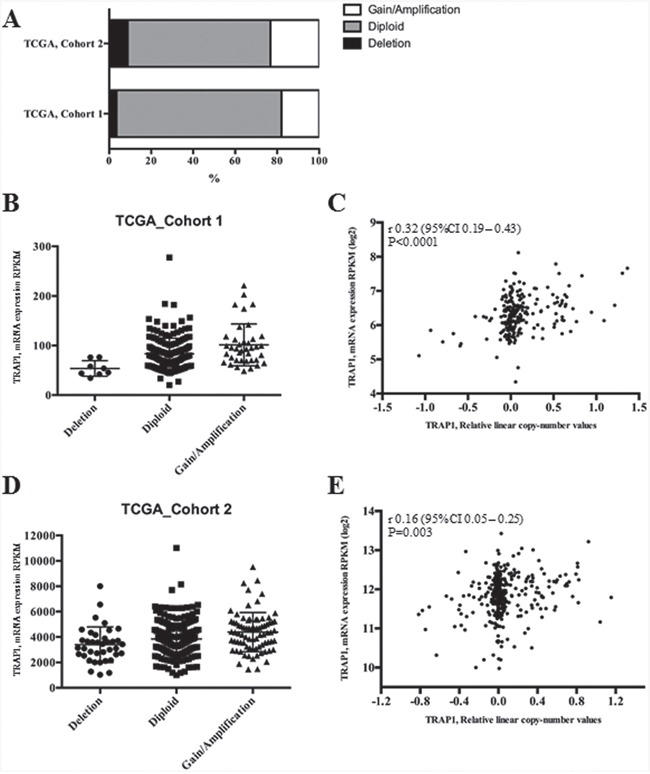
TRAP1 mRNA expression correlates with its gene copy number in TCGA datasets **A**. Percentages of CRCs with TRAP1 diploid genotype or with gain/loss of TRAP1 gene copy number in two public TCGA datasets. **B-E**. Distribution of TRAP1 mRNA expression according to its gene copy number (B and D) and statistical correlation between TRAP1 mRNA expression and its copy number linear level (C and E) in TCGA Cohort_1 (B-C) and Cohort_2 (D-E). B. Dunn's multiple comparisons test: Deletion vs. Diploid, p=0.0059; Deletion vs. Gain/Amplification; p=0.0001; Diploid vs. Gain/Amplification, p=0.0279. D. Deletion vs. Diploid, p=n.s.; Deletion vs. Gain/Amplification, p=0.0003; Diploid vs. Gain/Amplification, p=0.0152.

Since previous data in human ovarian carcinoma suggest that TRAP1 expression is dependent on genetic alterations regarding its gene copy number [22] the hypothesis that TRAP1 modulation in human CRC depends on transcriptional mechanisms was evaluated by analyzing the TCGA database. Data from two independent series (TCGA Cohorts 1 and 2) were used to establish the relationship between TRAP1 copy number and its mRNA expression (Figure 2). Interestingly, the vast majority of human CRCs exhibited a diploid TRAP1 genotype, with a cohort (ranging between 18 and 23% of cases) characterized by gain in TRAP1 copy number and a small subgroup (ranging between 3 and 9%) by TRAP1 gene shallow deletion (Figure 2A). The statistical analysis of both datasets showed a significant difference in TRAP1 mRNA expression levels according to TRAP1 copy number (Kruskal-Wallis, p=0.0001 in Cohort_1 and p=0.0003 in Cohort_2; Figure 2B–2C) and a significant correlation between TRAP1 mRNA expression and its copy number linear level [Spearman, R=0.32 (95% C.I. 0.19 – 0.43) p<0.0001 in Cohort_1, Spearman, R=0.16 (95% C.I. 0.05 – 0.25), p=0.003 in Cohort_2; Figure 2D–2E]. In addition, a statistically significant correlation was observed between TRAP1 copy number linear level and its protein expression in TGCA Cohort 2 (Spearman, R=0.31 (95% C.I. 0.1 – 0.48), p=0.004; Supplementary Figure 2). These data suggest that TRAP1 expression partially depends on transcriptional mechanisms in CRCs.

### TRAP1 is co-expressed with its client proteins

In further analysis, the expression levels of TRAP1 client proteins previously characterized by our group (i.e., F1ATPase, TBP7, IF2α, EF1G, IF4A, IF4E, EF1A, BRAF, AKT, 18kDa Sorcin, CDK1, MAD2, βCatenin) [11–13, 18, 20, 21] were comparatively evaluated, by immunoblot analysis, in the study cohort of 60 human CRCs (Supplementary Figure 1). Interestingly, Spearman Rank correlation test showed a statistically significant co-expression between TRAP1 and most of its client proteins (Table 2), being the statistical significance lacking only for F1ATPase and 18KDa Sorcin. In addition, a reciprocal co-expression was observed between the majority of TRAP1 client proteins (Supplementary Figure 3).

**Table 2:** Co-expression between TRAP1 and its network of client proteins

	TBP7	IF2α	EF1G	IF4A	IF4E	EF1A	BRAF	CDK1	MAD2	βCatenin
**Spearman R**	0.66	0.53	0.37	0.30	0.54	0.59	0.55	0.63	0.48	0.50
**95% C.I**.	0.47- 0.78	0.31-0.69	0.12-0.58	0.04-0.51	0.32-0.70	0.39-0.74	0.34-0.71	0.44-077	0.25-0.66	0.27-0.67
**p-value (two-tailed)**	< 0.0001	< 0.0001	0.0033	0.02	<0.0001	<0.0001	<0.0001	<0.0001	<0.0001	<0.0001

### TRAP1 proteome network predicts poor outcome in human metastatic CRCs

**Figure 3:**
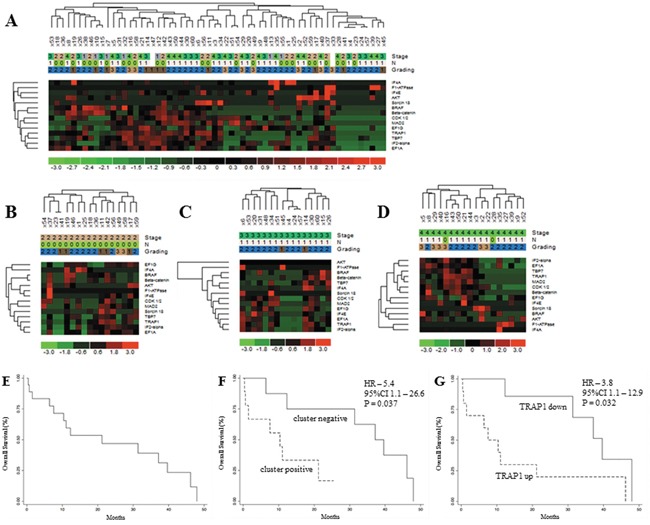
TRAP1 signature identifies metastatic colorectal carcinomas with different prognosis **A-D**. Hierarchical cluster analysis in 60 human CRCs (study cohort) (A) and stage II (B), III (C) and IV (D) tumors and its association with stage (TNM), lymph node involvement (N according to TNM classification) and grading. **E-F**. Kaplan Meier overall survival curves in the whole cohort of stage IV mCRCs (E) and according to the expression of TRAP1 and associated 6-protein signature (F) or TRAP1 itself **G**.

**Figure 4:**
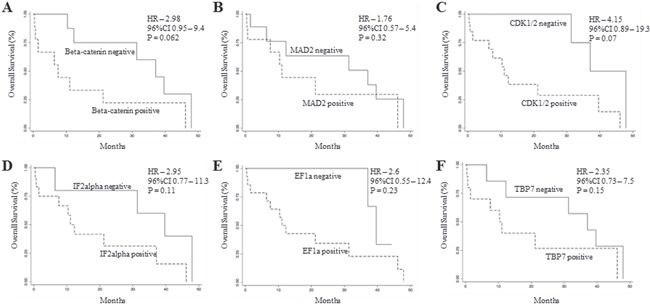
Overall survival analysis according TRAP1 client protein expression **A-F**. Kaplan Meier overall survival curves in stage IV mCRCs according to βCatenin (A), MAD2 (B), CDK1 (C), IF2α (D), EF1A (E) and TBP7 (F) positive/negative expression.

To analyze the prognostic significance of TRAP1 proteome, a hierarchical cluster analysis was used to obtain the segregation of our study cohort in independent clusters, according to the expression of TRAP1 client proteins. Interestingly, the concomitant expression of TRAP1 and eight clients proteins (concomitant up- versus down-regulation of BRAF, βCatenin, CDK1, MAD2, EF1G, EF1A, TBP7, IF2α) segregated CRCs in two major clusters not showing any association with TNM stage, lymph node involvement and grading (Figure 3A), neither with overall survival (data not shown). Thus, cluster segregation analysis was separately performed in TNM stage II, III and IV tumors (Figure 3B–3D). Noteworthy, TRAP1 proteomic signature segregated each subgroup in two independent clusters (Figure 3B–3D), and in stage IV mCRCs the concurrent positive/negative expression of TRAP1 and six of its client proteins (i.e., βCatenin, CDK1, MAD2, EF1A, TBP7, IF2α) allowed the segregation of tumors in clusters with different outcome. Indeed, Kaplan and Maier analysis showed significantly different OS curves in mCRCs with differential expression of either TRAP1 and associated 6-protein signature (HR 5.4; 95% C.I. 1.1-26.6; p=0.037; Figure 3E–3F) or TRAP1 itself (HR 3.8; 95% C.I. 1.1-12.9; p=0.032; Figure 3G). Interestingly, the upregulation of each TRAP1 client protein separately identified tumors with worst OS, but differences in OS curves did not reach the statistical significance (Figure 4A–4E).

**Figure 5:**
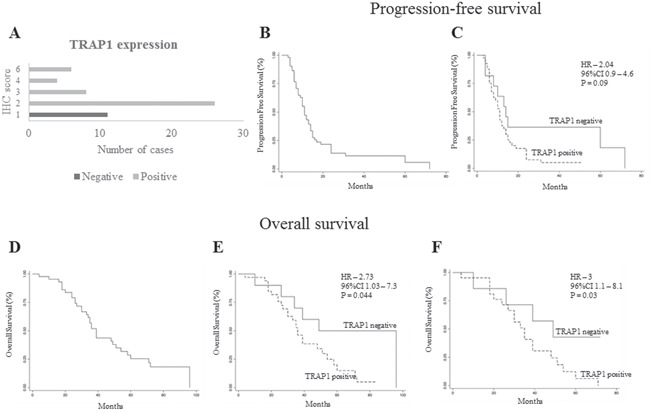
TRAP1 positive expression predicts poor prognosis in metastatic colorectal carcinomas **A**. Number of TRAP1-negative and positive tumors according to immunohistochemistry (IHC) score. **B-F**. Kaplan Meier progression-free survival (B-C) and overall survival (D-F) curves in the whole validation cohort of stage IV mCRCs (B and D) and according to TRAP1 expression (C and E) and in non-operable patients (F).

The prognostic significance of TRAP1 was further validated upon analysis of a validation cohort of 55 mCRCs treated with standard first line chemotherapy (oxaliplatin- or irinotecan-based chemotherapy ± anti-EGFR or anti-VEGF antibodies; Table 1). Thus, TRAP1 expression was quantified by immunohistochemistry, yielding 80% of cases with TRAP1 upregulation (Figure 5A). TRAP1 positive expression identified a cohort of mCRCs with a shorter PFS after first line chemotherapy compared to tumors with negative/low TRAP1 expression, even though this difference was not statistically different (HR 2.0; 95% C.I. 0.9-4.6; p=0.09; Figure 5B–5C). Noteworthy, OS analysis confirmed that tumors with high TRAP1 expression are characterized by a worst outcome compared to tumors with low TRAP1 expression (HR 2.7; 95% C.I. 1.0-7.3; p=0.044; Figure 5D–5E). In addition, the statistical difference in OS between TRAP1-positive and TRAP1-negative tumors was more significant after excluding patients who received surgical removal of liver/lung metastases (HR 3.0; 95% C.I. 1.1-8.1; p=0.03; Figure 5F), being lost in patients who received surgery for metastatic disease (data not shown). These data suggest that high TRAP1 expression predicts a more aggressive behavior and poor outcome in mCRCs.

### TRAP1 positive expression and its prognostic value are prevalent in left colon cancers

**Figure 6:**
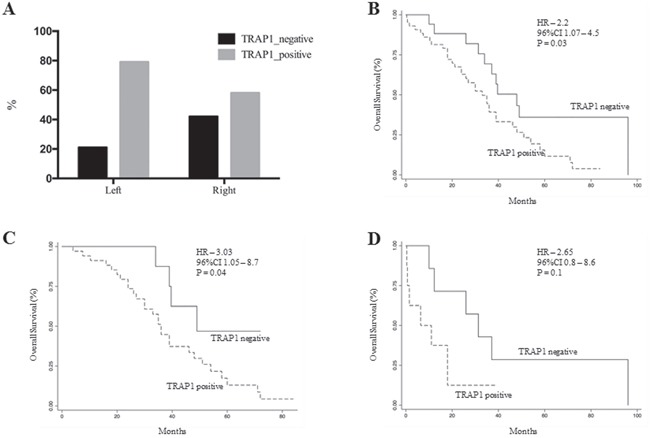
TRAP1 expression and its prognostic value are prevalent in left colon cancers **A**. Prevalence of TRAP1 upregulation in right versus left colon carcinomas. **B-D**. Kaplan Meier overall survival curves according to TRAP1 expression in the whole cohort of 73 stage IV mCRCs (B) and in left (C) versus right (D) colon cancers.

Recent evidence suggests that left and right colon cancers are distinct pathological entities with different biology and clinical response to therapy [23]. Thus, the relevance of TRAP1 prognostic value was evaluated in a combined cohort of 73 mCRCs, including all patients from the validation cohort and the subgroup of mCRCs from the study cohort. Interestingly, TRAP1 positive expression is enriched in left versus right CRCs, even though this difference is not fully significant (Figure 6A). In addition, overall survival curves confirmed the prognostic value of TRAP1 expression in the whole cohort (HR 3.0; 95% C.I. 1.1-8.1; p=0.03; Figure 6B) and in left colon CRCs (HR 3.03; 95% C.I. 1.05-8.7; p=0.04; Figure 6C). By contrast, the statistical significance in overall survival between TRAP1-positive and negative tumors is lost in right colon cancers (HR 2.65; 95% C.I. 0.8-8.6; p=0.1; Figure 6D), even though the lack of significance is likely due to the low number of right colon cancers in our series.

## DISCUSSION

Colorectal cancer is a heterogeneous disease respect to molecular profiles, drug response and clinical outcome [24]. Several attempts have been made to obtain gene expression-based classifications of human CRC and facilitate their clinical translation [2, 25]. However, at present, none of them produced clinically meaningful tools to enter daily clinical practice [3] and only the mutational status of specific genes (i.e., KRAS, NRAS and BRAF) is routinely used for treatment selection [24]. In such a context, we recently reported that i) the HSP90 molecular chaperone TRAP1 is upregulated in human CRCs [7], ii) its gene signature is associated to the stem like phenotype [18], and iii) its silencing results in the loss of the stem-like signature with acquisition of a more differentiated phenotype [18]. Since these evidences suggest that TRAP1 upregulation correlates with loss of differentiation and poor outcome in human CRC, this study was designed to address the clinical relevance of TRAP1 client protein network in human CRC. Our results show that: i) TRAP1 upregulation occurs at the transition between low- and high-grade adenomatous polyps, ii) human CRCs with high TRAP1 levels show a concomitant upregulation of TRAP1 client proteins network; iii) the upregulation of TRAP1 and its protein network predicts poor prognosis in mCRC, and iv) TRAP1 prognostic value is prevalent in left colon carcinomas.

It is intriguing that TRAP1 upregulation occurs at early stages of colorectal tumorigenesis, being already evident in high-grade adenomas and in *in situ* carcinomas. This evidence is consistent with previous studies showing that TRAP1 is upregulated in both dysplastic and non-dysplastic tissues of ulcerative colitis progressors, being its levels lower in colon tissues from ulcerative colitis non-progressors [26]. In addition, TRAP1-silenced colon carcinoma cells exhibited lack of clonogenic ability *in vitro* and tumor formation in mice xenografts [15], thus supporting the relevance of TRAP1 in early phases of colon carcinogenesis. It is also noteworthy that about 60% of human CRCs with high TRAP1 expression are characterized by the parallel upregulation of a network of proteins involved in several cell functions, critical for colon carcinogenesis [5, 10, 11, 13, 14, 18, 27, 28]. Thus, it is intriguing to speculate that TRAP1 upregulation may represent an early mechanism used by colon cancer cells to adapt to unfavorable environmental conditions and, thus, activate a number of pathways responsible for malignant transformation and tumor progression. This hypothesis is reinforced by previous evidences suggesting a crosstalk between TRAP1 and RAF/ERK pathway [13, 29] and TRAP1 regulation on Wnt/βCatenin signaling [18], two key pathways activated at early stages during colorectal carcinogenesis [31]. Altogether, these data support the concept that TRAP1 network should be acknowledged among signaling pathways responsible for colorectal transformation and progression.

Noteworthy, a small subgroup of colon cancers is characterized by TRAP1 levels below normal mucosa. The biological relevance of this observation is unclear, even though it is consistent with recent data showing that selected human malignancies (i.e., ovarian, cervical and renal carcinomas) are characterized by low TRAP1 expression [17] and that TRAP1 downregulation induces activation of oxidative metabolism and cisplatin resistance in ovarian carcinoma [30]. Thus, it is likely that human CRCs up/downregulate TRAP1 to adapt their fate to metabolic/environmental requirements, even though the significance of TRAP1 downregulation in selected CRCs is an issue that requires further investigation.

A major question is the mechanism used by cancer cells to modulate TRAP1 expression. The analysis of the TCGA database shows that the vast majority of human CRCs are characterized by a diploid TRAP1 genotype, with a subgroup, ranging between 21 and 32%, characterized by loss/gain of TRAP1 copy number. Interestingly, TRAP1 copy number variation correlates with its mRNA/protein expression, suggesting that genomic alterations may account for up/downregulation of TRAP1 expression only in a subgroup of human CRCs. Considering that TRAP1 protein level is upregulated in about 60% and downregulated in 13% of human CRCs, it is evident that posttranslational mechanism are also responsible for TRAP1 modulation. In such a perspective, a recent study demonstrated that S-nitrosylation is a major posttranslational modification responsible for regulation of TRAP1 expression in hepatocellular carcinoma [32]. Thus, further study are required to address the relationship between TRAP1 protein expression and gene copy number and the relevance of posttranslational modifications in TRAP1 regulation in human CRC.

The clinical relevance of TRAP1 signature in CRC was addressed by analyzing two independent cohorts of tumors. Indeed, TRAP1 expression itself as well as TRAP1 and its associated 6-protein signature predicted shorter OS in mCRCs in both the study cohort and in a separate cohort of 55 mCRCs treated with standard first-line chemotherapy. Noteworthy, TRAP1 prognostic value is significantly evident in patients who did not receive surgical resection of distant metastases, this supporting the hypothesis that the activation of TRAP1 network enhances the malignant potential and the clinical aggressiveness of human CRCs. Finally, TRAP1 capacity to predict poor prognosis were shown to be conserved in left CRCs, thus supporting the hypothesis that the upregulation of TRAP1 protein network may represent a further biological difference between right and left colon cancers.

These observations are consistent with previous studies suggesting that high TRAP1 expression correlates with lymph node metastases [8] and, together with ERCC1, with poor OS in mCRC treated with oxaliplatin/5-fluorouracil chemotherapy [9]. While our data did not confirm the association between TRAP1 and lymph node metastasis, they provide clear evidence that TRAP1 client protein network may be regarded as prognostic biomarker to drive clinical decisions in metastatic setting, even though several issues still needs to be addressed/confirmed in this perspective. Indeed, TRAP1 protein signature requires a further validation in a prospective larger clinical trial respect to specific therapy regimens (standard chemotherapy vs chemotherapy combined with molecular-targeted agents). In addition, the significance of TRAP1 prognostic value respect to primary tumor site (left versus right colon) requires further confirmation since our data do not rule out the hypothesis that the lack of full significance in right colon cancer OS curves may depend on the low number of right colon carcinomas in our series. Finally, it is important to establish whether the evaluation of specific client proteins together with TRAP1 provides additional prognostic information compared to TRAP1 alone.

In conclusion, this study provides the first comprehensive proteomic evaluation of TRAP1 protein network in colorectal tumors, showing the clinical relevance of its activation as a mechanism to coordinate a number of signaling pathway responsible for tumor progression and clinical aggressiveness.

## MATERIALS AND METHODS

### Study population

Two cohorts of, respectively, 60 and 55 human CRCs, 9 colorectal adenomatous polyps and in 11 *in situ* carcinomas were selected for this study. The study cohort of 60 CRCs at different TNM stages and corresponding normal, non-infiltrated peritumoral mucosa was obtained from the General Surgery Unit of the University of Foggia. Specimens were collected after surgical removal of tumors and immediately frozen in liquid nitrogen. The validation cohort of 55 paraffin-embedded metastatic CRCs (mCRCs) was obtained from the Pathology Unit of the IRCCS-CROB. Paraffin-embedded colorectal adenomatous polyps and *in situ* carcinomas were also obtained by the Pathology Unit of the IRCCS-CROB. Patient's characteristics, including clinical data referring to sex, age, tumor stage and grading, metastatic pattern and medical therapies are reported in Table 1. All patients gave their informed written consent to use biological specimens for investigational procedures. Tumor stage was classified according to TNM classification system [33].

### Immunohistochemistry

Four-μm serial sections from formalin-fixed and paraffin-embedded blocks were cut and mounted on poly-L-lysine-coated glass slides. Immunohistochemical analysis was performed using the Artisan Dako staining system (Dako Italia srl, Milano, Italy) and streptavidin-biotin horseradish peroxidase technique (LSAB-HRP), according to the best protocol for the antibody previously tested in our laboratory [14]. Mouse monoclonal anti-TRAP1 antibody (sc-73604) from Santa Cruz Biotechnology (Heidelberg, Germany) was used from immunostaining. Sections were counterstained with type-II-Gill's haematoxylin, dehydrated with ethanol and permanently coverslipped. Results of the immunohistochemical staining were evaluated separately by two observers particularly trained for colorectal pathology and completely blind to the histological diagnosis. The inter-rater reliability between the two investigators examining the immunostained sections was assessed by the Cohen's K test, yielding K values >0.70 in all instances. Immune-stained cells were counted in at least 10 High Power Fields (HPF) analyzed with an optical microscope (Olympus BX53; Olympus Italia, Milan, Italy) at 40x magnification. TRAP1-positive staining was defined as perinuclear (mitochondrial) staining in tumor tissue. The number of TRAP1-expressing tumor cells was estimated as a mean percentage of total number of cells per section and grouped according to the percentage of positive cells: 0 (no staining), 1 (1%-33%), 2 (34%-66%) and 3 (67%-100%). The intensity of TRAP1 staining was graded as 0 (no staining), 1 (weak/moderate), 2 (strong). A combined numeric IHC score was calculated as the product of staining intensity and percentage of stained cells [14]. TRAP1 staining was interpreted as positive in case of a product > 1.

### Immunoblot analysis

Immunoblot analysis was performed as previously reported [13]. The following mouse monoclonal antibodies from Santa Cruz Biotechnology (Heidelberg, Germany) were used: anti-TRAP1 (sc-73604), anti-BRAF (sc-5284), anti-TBP7 (sc-166003), anti-eIF2α (sc-133132), anti-F1ATPase (ATP5B subunit; sc-58619), anti-CDK1 (sc-53219), anti-MAD2 (sc-393188), anti-glyceraldehyde-3-phosphate dehydrogenase (GAPDH, sc-69778). The following antibodies were also used: mouse monoclonal anti-eEF1A (#05-235) from Millipore; rabbit monoclonal anti-eIF4E (#C46H6), rabbit monoclonal anti-eIF4A (#C32B4), rabbit polyclonal anti-AKT (#9272), rabbit polyclonal anti-βCatenin (#9562) from Cell Signaling Technology, rabbit polyclonal anti-EEF1G (#NB100-2263) from Novus Bio. Rabbit polyclonal anti-Sorcin antibody was kindly provided by Prof. E Chiancone (University of Rome “La Sapienza”). Levels of specific proteins were quantified by densitometric analysis using the Quantity One 4.5 software (BioRad Laboratories GmbH) and considered positive in case of values ≥ 2 times increase compared to normal mucosa.

### TCGA database analysis

TCGA Cohort_1http://www.cbioportal.org/study?id=coadread_tcga and Cohort_2 (Provisional) (level 3 data) were used to correlate TRAP1 mRNA expression performed by total RNA sequencing and protein by mass spectrometry with Copy Number Alterations data performed by array methods and calculated with GISTIC statistics. Kruskal-Wallis test was used to compare gene expression between more than two groups (Deletion, Diploid and Gain/Amplification). Post-hoc Dunn's test was performed to evaluate multiple comparison. The Sperman R (with 95% confidence interval) was calculated to evaluate correlation of gene expression with relative linear copy-number values.

### Statistical analysis

The Sperman R (with 95% confidence interval) was calculated to evaluate correlation between TRAP1 expression and its client proteins in the study cohort. Unsupervised analysis of TRAP1 expression and its client protein was performed to evaluate patients clustering and grouping of signature. Heatmap and hierarchical clusters were generated using dChip (DNA-Chip Analyzer software), with inverse correlation as distance metric and average linkage method.

Progression Free Survival (PFS) and OS curves were estimated and plotted with the Kaplan-Meier method. Hazard ratio (HR) and the corresponding two-sided 95% confidence interval (95% CI) were estimated by Cox proportional hazards models. Statistical significance was defined as p <0.05. Analyses were performed using package R version 3.2.3 (The R Project for Statistical Computing). Continuous data were reported as means and standard deviations or medians and range, meanwhile categorical data were reported as counts and percentages.

## SUPPLEMENTARY MATERIALS FIGURES AND TABLES


