# Supplementary material for: TRAP1 protein signature predicts outcome in human metastatic colorectal carcinoma
Source: Oncotarget. 2017 Feb 3;8(13):21229–40. doi: 10.18632/oncotarget.15070 (PMC5400579; doi:10.18632/oncotarget.15070)
Supplement: Supplementary file 1 [file oncotarget-08-21229-s001.pdf]

TRAP1 protein signature predicts outcome in human metastatic colorectal carcinoma

SUPPLEMENTARY FIGURES AND TABLE

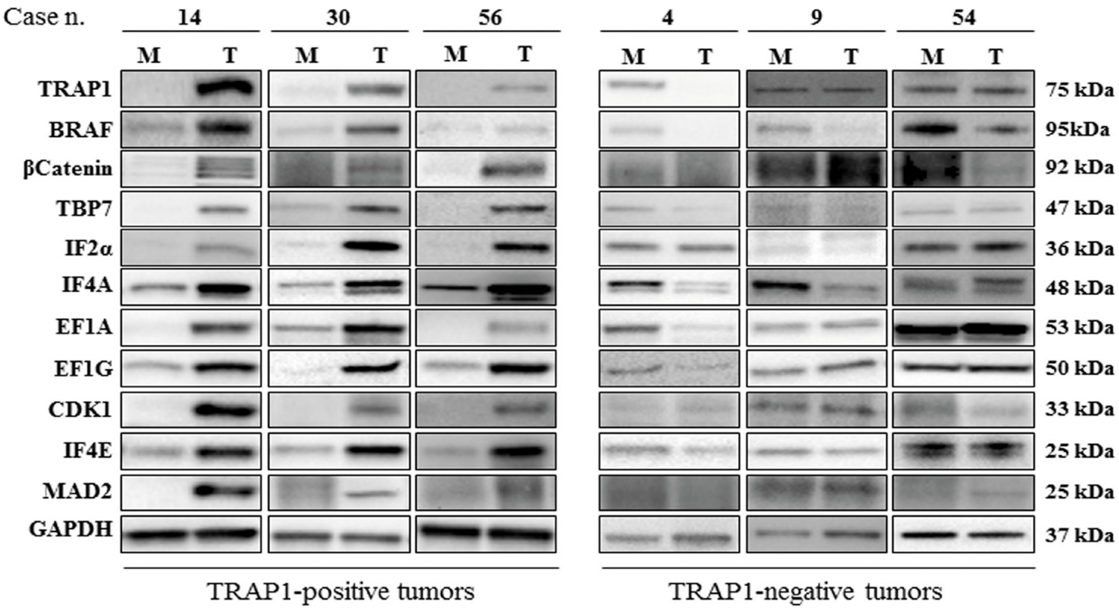

Supplementary Figure 1: Co-expression of TRAP1 and its client protein network. Immunoblot analysis of TRAP1 and its client protein network in 6 representative cases of the study cohort.

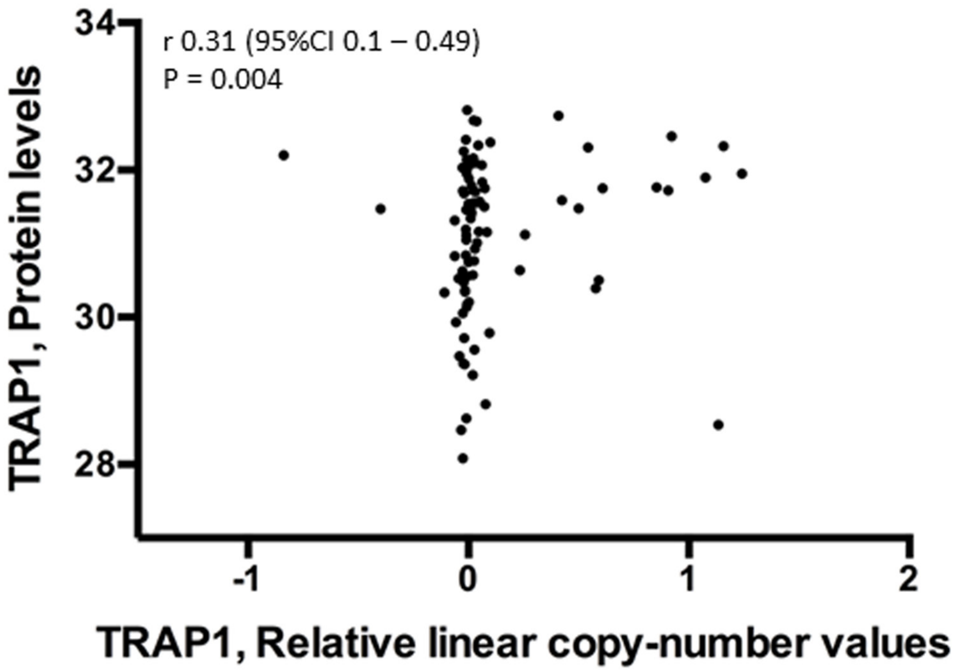

Supplementary Figure 2: TRAP1 protein expression correlates with its gene copy number. Correlation between TRAP1 protein expression and its copy number linear level in TCGA Cohort_2.

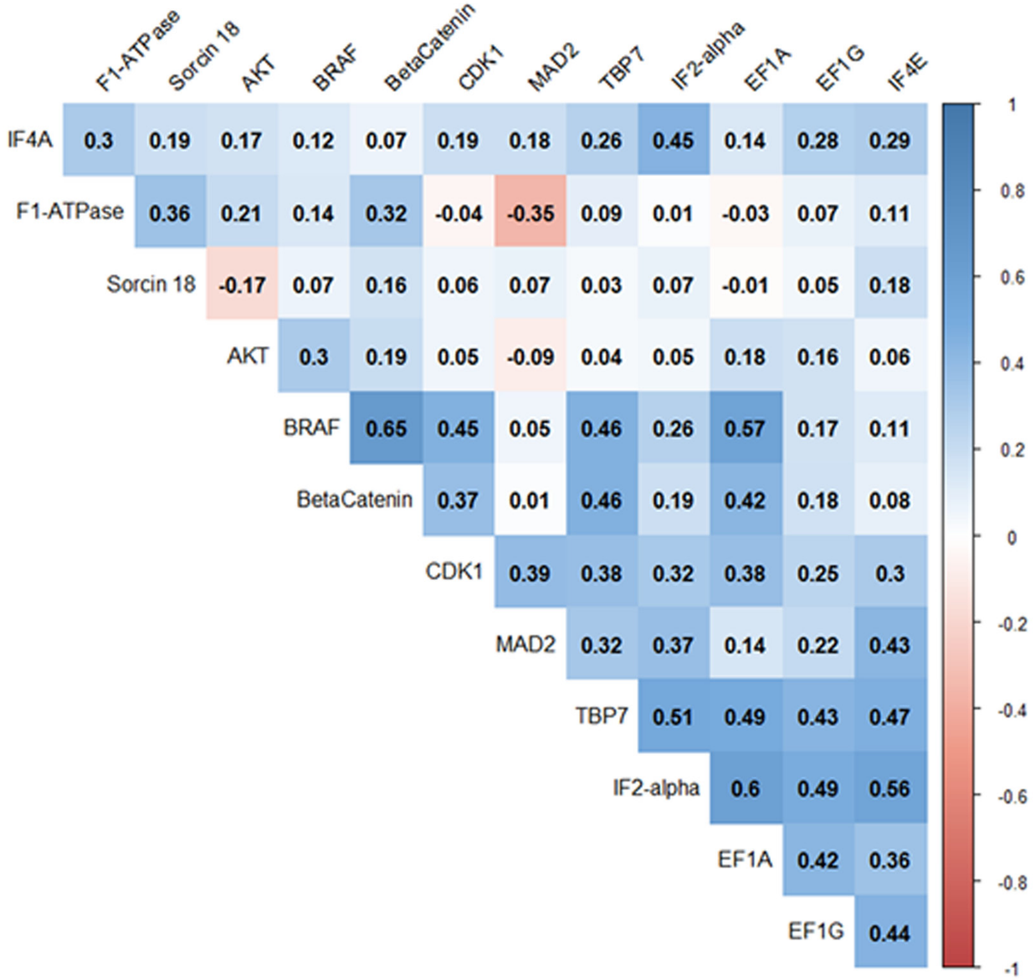

Supplementary Figure 3: Reciprocal correlation between TRAP1 client proteins. Correlation plot reporting Spearman r coefficients between TRAP1 network proteins.

Supplementary Table 1: TRAP1 expression in 60 human colorectal carcinomas and its distribution according to major pathological covariates

**TRAP1** (60 cases)		
• Mean (±S.D.)	13.4 ± 22.23	
• Median (range)	3.15 (0.11 - 93.48)	
**TNM**	**Median (range)**	p-value
• I	2.4 (0.4 - 48.1)	
• II	3.7 (0.33 - 93.5)	
• III	4.005 (0.1 - 82.3)	0.83
• IV	2.44 (0.18 - 67.6)	
**Grade**		
• 1	9.33 (0.7 - 93.48)	
• 2	3.27 (0.11 - 82.24)	0.35
• 3	2.55 (0.9 - 67.6)	
**Lymph nodes**		
• N negative	3.72 (0.33 - 93.48)	
• N positive (1-3)	3.4 (0.16 - 79.6)	0.47
• N positive (>3)	2.44 (0.11 - 36.74)	
